# Retraction: Suppression of Neuroinflammatory and Apoptotic Signaling Cascade by Curcumin Alone and in Combination with Piperine in Rat Model of Olfactory Bulbectomy Induced Depression

**DOI:** 10.1371/journal.pone.0215680

**Published:** 2019-04-15

**Authors:** The PLOS ONE Editors

Concerns have been raised about results presented in several figures of this article [1]. Specifically, it was noted that the results in the following graph pairs show unexpectedly high degrees of correlation if one considers fold changes across groups:

- Fig 1 Sucrose water intake and Fig 2 Immobility time
- Fig 4 Complex III and Fig 6 TNFα
- Fig 4 Complex IV and Fig 5 Serum corticosterone
- Fig 5 Serum corticosterone and Fig 8 BDNF

A member of *PLOS ONE*’s Editorial Board and an external reviewer with statistical expertise expressed concerns that the same fold changes were observed between the indicated datasets for all groups, and advised that the degree of correlation between variates appears to exceed considerably what would be expected in such experiments given the assays used, the number of animals per group, and that different groups of animals were used for the different experiments.

The corresponding author commented that the results in these figures are from different experiments expected to yield opposite results. The underlying data supporting the results are not available.

The *PLOS ONE* Editors retract this article in light of concerns about the validity of the data reported in these figures.

PR, AK, and SG do not agree with retraction.
